# Salivary Cortisol Response After Ropivacaine–Dexamethasone Administration: A Randomized Clinical Trial

**DOI:** 10.3390/ph19060930

**Published:** 2026-06-12

**Authors:** Simona M. Stojanović, Nikola B. Burić, Miloš S. Kostić, Kristina N. Burić, Branislava B. Stojković, Milan S. Spasić, Miloš Tijanić, Miloš Trajković, Rodoljub Jovanović, Milica S. Petrović

**Affiliations:** 1 Department of Oral Surgery, Clinic for Dental Medicine, Faculty of Medicine, University of Niš, 18108 Niš, Serbia; simona.stojanovic@medfak.ni.ac.rs (S.M.S.); nikola.buric@medfak.ni.ac.rs (N.B.B.); milan.s.spasic@gmail.com (M.S.S.); milos.tijanic@medfak.ni.ac.rs (M.T.); rodoljub1997@gmail.com (R.J.); 2 Department of Immunology, Faculty of Medicine, University of Niš, 18108 Niš, Serbia; milos.kostic@medfak.ni.ac.rs; 3 Department of Oral Surgery, Faculty of Medicine, University of Niš, 18108 Niš, Serbia; kristina.buric@yahoo.com; 4 Department of Preventive and Pediatric Dentistry, Clinic for Dental Medicine, Faculty of Medicine, University of Niš, 18108 Niš, Serbia; branislava.stojkovic@medfak.ni.ac.rs; 5 Department of Maxillofacial Surgery, Clinic for Dental Medicine, Faculty of Medicine, University of Niš, 18108 Niš, Serbia; dr.mtrajkovic@yahoo.com; 6 Department of Oral Medicine and Periodontology, Faculty of Medicine, University of Niš, 18108 Niš, Serbia

**Keywords:** salivary cortisol, surgical stress, third molar surgery

## Abstract

**Background:** This randomized parallel-group clinical study evaluated salivary cortisol as a biomarker of perioperative stress response during surgical extraction of impacted mandibular third molars performed under local anesthesia administration of ropivacaine and dexamethasone. **Methods:** The trial was registered in the ISRCTN registry (ISRCTN87752106). Ninety patients undergoing impacted mandibular third molar surgery were randomly assigned (1:1:1; *n* = 30/group) to receive inferior alveolar nerve block with 0.5% ropivacaine plus dexamethasone (R+D group), 0.5% ropivacaine (R group), or 0.5% bupivacaine (B group). Salivary cortisol as the primary outcome was measured at 15 min before anesthesia, and 15 min and 24 h postoperatively. Preoperative psychological stress was assessed using the Revised Norman Corah Dental Anxiety Scale. **Results:** No significant differences were observed between groups in preoperative anxiety (*p* = 0.890) or baseline salivary cortisol levels (*p* = 0.984). Significant intergroup differences in cortisol levels were observed at 15 min (*p* = 0.002) and 24 h postoperatively (*p* = 0.001). Cortisol levels at 15 min postoperatively were significantly lower in the R+D group compared to the R (*p* = 0.001) and B groups (*p* = 0.004) and after 24 h (R+D vs. R: *p* = 0.005; R+D vs. B: *p* < 0.001). **Conclusions:** The ropivacaine–dexamethasone administration significantly reduced perioperative salivary cortisol levels compared to ropivacaine alone or bupivacaine alone during impacted mandibular third molar surgery. This modulation of the neuroendocrine response likely results from dexamethasone-induced suppression of hypothalamic–pituitary–adrenal axis and the improved analgesic effects of this combination. The observed results may contribute to improved physiological stability, postoperative recovery and the clinical benefit of this anesthetic approach in oral surgery.

## 1. Introduction

Surgical removal of impacted mandibular third molars is one of the most frequently performed procedures in oral and maxillofacial surgery [1]. This procedure is associated with postoperative pain, inflammation, and activation of perioperative systemic neuroendocrine stress responses [2]. Postoperative pain results from inflammatory tissue injury and immune activation, leading to release of proinflammatory mediators that stimulate nociceptive pathways and prostaglandin synthesis [3]. The most pronounced nociceptive response typically occurs several hours after local anesthesia wears off, with pain peaking within the first postoperative day [4].

Long-acting local anesthetics such as ropivacaine and bupivacaine act via voltage-gated sodium channel blockade and are commonly used to prolong intraoperative anesthesia and improve postoperative analgesia [5]. Beyond their anesthetic properties, amide local anesthetics such as ropivacaine and bupivacaine have been shown to exert direct immunomodulatory effects by inhibiting leukocyte metabolic activation and pro-inflammatory mediator release, while also providing cellular protection against inflammatory injury through mechanisms involving mitochondrial potassium channels and reduced endothelial damage [6,7]. These findings suggest that the pharmacological actions of local anesthetics extend beyond sodium channel blockade to include anti-inflammatory and cytoprotective effects. Compared to bupivacaine, ropivacaine possesses a more favorable safety profile, including lower cardiotoxicity and arrhythmogenic potential [8,9]. Ropivacaine exhibits anti-inflammatory and analgesic properties through inhibition of leukocyte migration and modulation of inflammatory pathways [10,11,12] and demonstrates lower cytotoxicity in human cell models compared to bupivacaine [13], which supports increasing use of ropivacaine in oral surgery.

Previous studies have suggested that dexamethasone added to ropivacaine for inferior alveolar nerve block prolongs perioperative anesthesia and improves postoperative analgesia during mandibular third molar surgery [14]. The mechanisms underlying these effects are likely multifactorial, involving both peripheral and systemic pathways. Improved anesthetic approaches may modulate perioperative neuroendocrine responses by influencing inflammatory signaling pathways, thereby contributing to improved postoperative recovery [15].

Mandibular third molar surgery, similarly to other surgical procedures, induces a perioperative systemic stress response involving neuroendocrine, metabolic, inflammatory, and psychological pathways activated to maintain homeostasis [16]. Cortisol, a steroid hormone produced by the adrenal cortex, represents a key mediator of the stress response through activation of the hypothalamic–pituitary–adrenal (HPA) axis and influences multiple physiological systems, including cardiovascular, metabolic, neurological, and musculoskeletal functions. It is also one of the most widely studied salivary biomarkers of stress and anxiety [17]. Cortisol secretion is regulated by the HPA axis, where stress stimulates the hypothalamus to release corticotropin-releasing hormone (CRH), which in turn induces secretion of adrenocorticotropic hormone (ACTH) from the anterior pituitary gland. ACTH subsequently stimulates the adrenal cortex to release cortisol. Increased circulating cortisol levels exert negative feedback on both the hypothalamus and pituitary gland, thereby suppressing further CRH and ACTH release and maintaining hormonal homeostasis. Under physiological conditions, cortisol follows a circadian rhythm characterized by a pronounced peak approximately 30 min after awakening, followed by a gradual decline throughout the day and lowest levels during the late evening and night [18,19]. Salivary cortisol reflects biologically active free cortisol and enables non-invasive assessment of perioperative neuroendocrine stress responses. Because free cortisol readily diffuses into saliva, salivary cortisol is considered a reliable biomarker of perioperative stress and HPA axis activity [20]. Mean salivary cortisol concentrations are approximately 8.4 ng/mL in the morning and 2.2 ng/mL in the evening [21].

Dexamethasone is a synthetic analogue of cortisol approximately 25–40 times more potent in terms of anti-inflammatory activity [22]. Its anti-inflammatory effects are primarily mediated through genomic mechanisms. Following binding to intracellular glucocorticoid receptors, the receptor–drug complex translocates to the nucleus and modulates gene transcription, resulting in increased expression of anti-inflammatory mediators and suppression of proinflammatory cytokines [23]. Dexamethasone additionally inhibits leukocyte migration and suppresses prostaglandin and leukotriene synthesis through lipocortin-mediated phospholipase A2 inhibition [23,24]. Although the systemic effects of dexamethasone are predominantly delayed and genomically mediated through HPA axis suppression, dexamethasone may also exert rapid non-genomic actions, including modulation of vascular tone and increased vascular responsiveness to catecholamines [23,25]. These properties support its use as an adjuvant to long-acting local anesthetics for improved perioperative pain control.

Despite evidence supporting the analgesic and anti-inflammatory effects of the ropivacaine–dexamethasone combination, limited data exist regarding its direct influence on perioperative HPA axis activation during mandibular third molar surgery, particularly when assessed through salivary cortisol dynamics.

Accordingly, the primary aim of this study was to evaluate the effect of the ropivacaine–dexamethasone combination for inferior alveolar nerve block on perioperative salivary cortisol levels compared with ropivacaine and bupivacaine alone during mandibular third molar surgery. Secondary outcomes included assessment of preoperative stress and evaluation of postoperative anesthetic and analgesic efficacy of this combination.

## 2. Results

**Figure 1:**
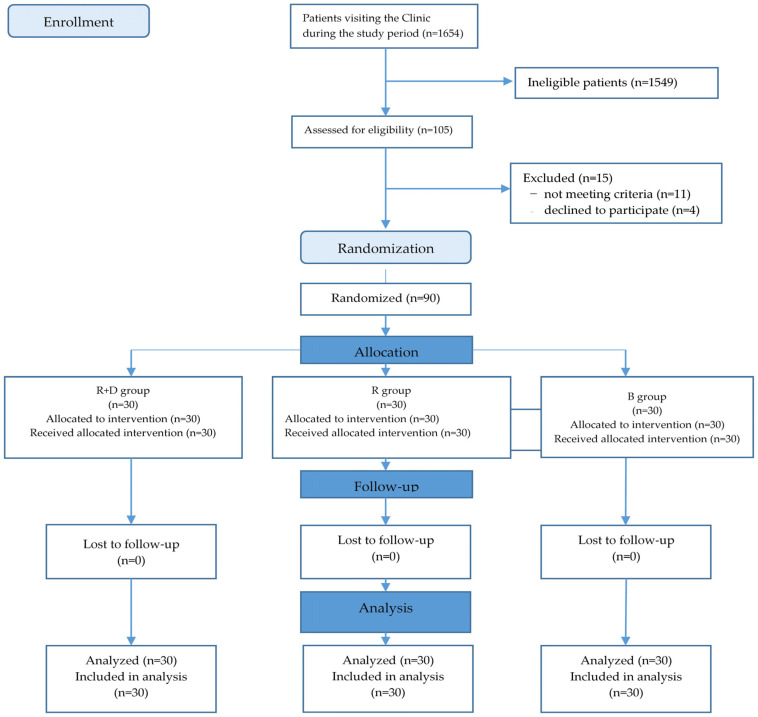
Flow chart diagram of patient allocation.

A total of 90 patients were included and randomized into three study groups (n = 30 per group). No participants were lost to follow-up, and all patients were included in the final analysis according to the assigned groups (Figure 1).

Baseline demographic and clinical characteristics are presented in Table 1. The study groups were comparable in age (*p* = 0.067), gender distribution (*p* = 0.488), surgery duration (*p* = 0.098), and preoperative dental anxiety scores (*p* = 0.890) (Table 1).

Duration of conduction anesthesia, duration of postoperative analgesia, and analgesic consumption differed significantly among groups (Table 1). Patients in the R group required a higher number of analgesics within 24 h compared to the R+D and B groups (*p* < 0.001) (Table 1).

Perioperative salivary cortisol levels showed a significant group × time interaction (*p* < 0.001) with a large effect size (partial η^2^ = 0.200). Preoperative cortisol levels were comparable between groups (*p* = 0.984). Cortisol levels decreased significantly in the R+D group (*p* < 0.001), whereas no significant changes were observed in the R (*p* = 0.071) or B group (*p* = 0.076). Pairwise comparisons confirmed that cortisol levels at time 1 were significantly higher than at time 3 (mean difference 3.81 nmol/L, 95% CI 0.86–6.76, *p* = 0.007), while no significant difference was observed between time 1 and time 2 (mean difference 1.04 nmol/L, 95% CI –1.60 to 3.68, *p* = 1.000).

**Figure 2:**
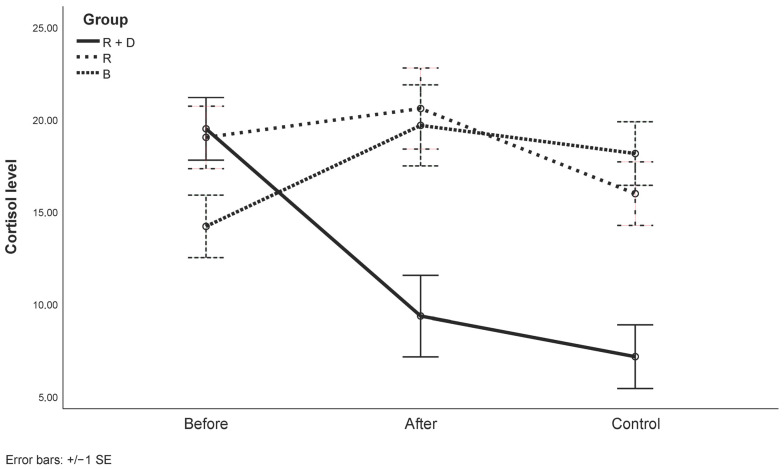
Changes in salivary cortisol levels between the study groups.

Statistically significant differences were observed between groups 15 min postoperatively (*p* = 0.002) as well as 24 h (*p* = 0.001). Postoperative cortisol levels were significantly lower in the R+D group compared to the R (*p* = 0.001) and B groups (*p* = 0.004) (Table 2, Figure 2).

Cortisol levels were significantly lower in the R+D group compared to the R (*p* = 0.005) and B groups (*p* < 0.001) 24 h after surgery (Table 2, Figure 2).

No serious adverse events were observed during the study. In the R+D group, one positive aspiration occurred during administration of the inferior alveolar nerve block, without further complications. In the R group, one patient reported dizziness that persisted for approximately 2 days and resolved spontaneously. In the B group, one patient developed a postoperative hematoma, which resolved within a few days without additional treatment.

## 3. Discussion

Perioperative stress represents a complex physiological and psychological response associated with oral surgical procedures, with salivary cortisol serving as an established biomarker of hypothalamic–pituitary–adrenal (HPA) axis activation [16]. Previous studies demonstrated that effective analgesia and anti-inflammatory effects of local anesthetics may contribute to modulation of postoperative neuroendocrine responses [2,26]. In the present study, patients who received the ropivacaine–dexamethasone (R+D) combination for inferior alveolar nerve block exhibited lower salivary cortisol levels compared to patients treated with local anesthetics alone, i.e., the R and B groups. These findings should be interpreted cautiously, considering the known suppressive effects of dexamethasone on HPA axis activity and cortisol secretion. Similar to previous research [14], this study found that the ropivacaine–dexamethasone combination prolonged anesthesia and postoperative analgesia while reducing postoperative analgesic consumption. The ropivacaine–dexamethasone combination was associated with lower early and delayed postoperative salivary cortisol levels, supporting the potential clinical utility of dexamethasone as an adjuvant to ropivacaine in mandibular third molar surgery.

Preoperative anxiety may increase stress perception and pain sensitivity, thereby influencing the overall perioperative experience [27]. Brand et al. [28] reported an association between dental anxiety and cortisol excretion prior to dental treatment. In the present study, preoperative dental anxiety scores, as an indicator of subjective psychological response, ranged from mild to moderate and were correlated with increased salivary cortisol levels across all groups prior to the initiation of the surgical procedure, suggesting activation of both psychological and physiological components of the perioperative stress response. Liau et al. [29] also demonstrated that increased dental anxiety is associated with exaggerated physiological stress responses during dental procedures. These findings are consistent with the present results and further support the distinction between anxiety as a subjective psychological state and stress as a measurable physiological response. Local anesthetics containing vasoconstrictors may enhance cardiovascular responses in anxious patients, so the present study utilized long-acting local anesthetics without vasoconstrictors. In addition, all participants underwent impacted mandibular third molar surgery for the first time, minimizing the potential influence of previous surgical experience on stress response.

Studies by Gadicherla et al. [30], Miller et al. [31], Kumari et al. [32], and others [33,34] consistently demonstrate perioperative activation of the hypothalamic–pituitary–adrenal (HPA) axis, reflected by increasing salivary cortisol levels before and after tooth extraction. Opaleye et al. demonstrated that benzodiazepine premedication has a limited effect on perioperative salivary cortisol levels during third molar surgery [35]. In contrast, the findings of the present study suggest that local administration of dexamethasone in combination with ropivacaine may contribute to modulation of salivary cortisol dynamics. Anesthetic and pharmacological agents may play a significant role in modulating neuroendocrine stress pathways and attenuating the physiological response to surgical trauma through effects on nociceptive transmission, inflammatory signaling, and activation of the hypothalamic–pituitary–adrenal (HPA) axis. Amide local anesthetics such as ropivacaine and bupivacaine exert immunomodulatory and anti-inflammatory effects beyond sodium channel blockade, including inhibition of leukocyte activation and inflammatory mediator release [6,7]. Dexamethasone-induced systemic suppression of the hypothalamic–pituitary–adrenal (HPA) axis is typically delayed, occurring several hours after, with maximal effects approximately 24 h after administration [36], following a dose (8 mg) twice as high as that used in the present study (4 mg). The lower cortisol levels observed in the immediate postoperative period (15 min postoperatively) may also reflect indirect effects such as reduced nociceptive input and improved perioperative analgesia. Perioperative cortisol changes observed 15 min after surgery likely reflect a combination of time-dependent pharmacodynamic effects, synergistic anti-inflammatory actions, and reduced nociceptive input associated with dexamethasone and ropivacaine. Glucocorticoids exert their effects via both delayed genomic and fast non-genomic actions [23,25]. Genomic actions include suppression of proinflammatory cytokine expression, inhibition of leukocyte migration, and reduced release of inflammatory mediators, while non-genomic effects involve rapid modulation of intracellular signaling and neuronal excitability. Experimental studies have shown that glucocorticoids can increase the expression of potassium channels, leading to reduced neuronal excitability [37]. This means that dexamethasone in combination with ropivacaine, which also acts through ion channels, could contribute to a prolonged perineural analgesic effect and potential attenuation of neuroendocrine activity. Preclinical data indicate that dexamethasone reduces bupivacaine-induced neuronal injury through activation of PI3K/Akt-dependent survival pathways, suggesting a potential mechanism underlying its perineural analgesic benefits [38]. Dexamethasone may reduce local blood flow and exert vasoconstrictive effects at the site of administration [39], limiting systemic absorption of local anesthetics and prolonging their neural and perineural retention. The non-genomic, membrane-associated actions of glucocorticoids may play a role in modulating neuronal excitability and local inflammatory responses, potentially related to the prolonged effects observed with perineural dexamethasone administration [37,38,39,40]. Dexamethasone is highly lipophilic and may modulate the perineural microenvironment through anti-inflammatory and vascular effects, thereby contributing to prolonged local anesthetic action. Supporting a peripheral interaction, Jaeger et al. demonstrated that perineural dexamethasone combined with ropivacaine for saphenous nerve block significantly prolonged anesthesia compared to ropivacaine alone [41]. These effects may be explained by prolonged suppression of nociceptive input by ropivacaine, together with the anti-inflammatory and glucocorticoid non-genomic and genomic mediated actions of dexamethasone. The mild vasoconstrictive effect of dexamethasone may reduce vascular absorption and further prolong anesthetic action. Together, these mechanisms may contribute to improved perioperative pain control and attenuation of perioperative neuroendocrine responses, including salivary cortisol reduction.

From an endocrinological perspective, interpretation of dexamethasone-mediated modulation of salivary cortisol requires consideration of glucocorticoid potency and HPA axis suppressive capacity. Based on glucocorticoid equivalence, 8 mg of dexamethasone corresponds approximately to 200–213 mg of cortisol in terms of glucocorticoid activity and potential suppression of HPA axis function [42]. Accordingly, the 4 mg dose administered in the present study corresponds to an estimated cortisol-equivalent activity of approximately 100–106 mg, representing a relatively modest systemic glucocorticoid exposure. In contrast, severe physiological stress has been associated with endogenous cortisol secretion reaching approximately 200–300 mg [43]. Therefore, although the 4 mg dexamethasone dose used in this study may have contributed to partial attenuation of perioperative cortisol responses, higher doses may be required to achieve more profound suppression of stress-induced HPA axis activation in patients exhibiting pronounced physiological stress reactions.

Prolonged elevation of cortisol levels may adversely affect oral wound healing by suppressing immune function, reducing fibroblast activity and collagen synthesis, and disrupting the balance of inflammatory responses. Additionally, elevated cortisol levels may impair bone remodeling by inhibiting osteoblast activity and interfering with mineralization processes, potentially compromising postoperative bone healing following extraction of impacted mandibular molars [44,45,46]. Local anesthetics (LAs) exhibit anti-inflammatory effects at both humoral and cellular levels [47], with some studies suggesting efficacy comparable to certain steroidal and non-steroidal anti-inflammatory approaches [2]. Addition of dexamethasone may enhance and prolong the analgesic and anti-inflammatory effects of ropivacaine. Perioperative anesthetic and analgesic management may influence neuroendocrine and immune responses to surgical trauma. Adequate pain control and attenuation of nociceptive input may reduce activation of stress-related inflammatory and hormonal pathways, potentially contributing to improved postoperative recovery. In this context, lower perioperative and prolonged postoperative salivary cortisol levels, together with greater physiological stability, may support more favorable recovery dynamics and enable earlier return to normal daily activities following impacted mandibular third molar surgery.

Limitations: This study has several limitations. The sample size was relatively small, which may limit the generalizability of the findings. The results are applied to healthy patients undergoing standardized surgical extraction of impacted mandibular third molars.

Changes in salivary cortisol levels should be interpreted cautiously. They reflect both stress attenuation and the pharmacological effects of dexamethasone on the HPA axis. Lower postoperative cortisol levels may indicate a more favorable physiological response, but the clinical relevance of this finding requires further investigation.

The selected timing of cortisol sampling, particularly in the early postoperative period, may not fully reflect peak cortisol concentrations or the complete temporal dynamics of surgical stress and dexamethasone action.

## 4. Materials and Methods

### 4.1. Study Design and Participants

This prospective, randomized, parallel-group, controlled clinical study was designed to compare three local anesthetic regimens in patients undergoing surgical extraction of impacted mandibular third molars. The study population consisted of patients treated at the Clinic of Dental Medicine, Faculty of Medicine, University of Niš, Department of Oral Surgery, between 15 July 2019 and 15 July 2022. Eligible participants were patients scheduled for surgical extraction of horizontally impacted mandibular third molars who had no previous history of mandibular third molar extraction. Prior to enrollment, all participants received detailed verbal and written information regarding the study objectives, study procedures, medications used, and measures undertaken to ensure confidentiality and protection of personal data. Written informed consent was obtained from all participants before inclusion in the study. The study protocol was approved by the Ethics Committee of the Faculty of Medicine, University of Niš, Serbia (Approval No. 12-7476-2/2; approved on 1 July 2019). The study was initiated following Ethics Committee approval and was retrospectively registered in the ISRCTN registry (ISRCTN87752106; Salivary cortisol as a biomarker of surgical stress and clinical outcomes following third molar surgery. Available from: https://doi.org/10.1186/ISRCTN87752106, 11 May 2026) in accordance with institutional regulatory requirements. The study was retrospectively registered because, according to institutional regulations of our country, at the time of study initiation, commencement of the clinical study was permitted following approval by the institutional Ethics Committee, without a prior requirement for prospective trial registration. The trial was subsequently registered using the same study objectives, methodology, and protocol details that had previously been submitted to and approved by the Ethics Committee. The study was conducted in accordance with the ethical principles outlined in the Declaration of Helsinki and reported in compliance with the CONSORT guidelines [48]. Participants were randomly allocated in a 1:1:1 ratio to three study groups. The random allocation sequence was generated using computer-based randomization software by an independent investigator not involved in participant recruitment, treatment, or outcome assessment. Allocation concealment was ensured using sequentially numbered, opaque, sealed envelopes. Participants and the operating oral surgeon as well as other staff were blinded to group allocation. An independent dental nurse, not involved in clinical procedures or outcome assessment, prepared all anesthetic solutions in identical 5 mL syringes with equal volume and indistinguishable appearance. Syringes were labeled with anonymized study codes only. Sequentially numbered, opaque, sealed envelopes were used for allocation concealment and were opened in numerical order at the time of participant enrollment, immediately prior to intervention. Outcome assessors remained blinded to group allocation until completion of the study. All injections were administered using the same anesthetic technique, needle type, and injection protocol. Group assignment remained concealed from both the participants and the operating oral surgeon throughout the entire study period and outcome assessment, thereby ensuring a double-blind study design. A total of 105 patients were assessed for eligibility, of whom 90 were randomized and equally allocated into three study groups (n = 30 per group). No participants were lost to follow-up, and all randomized patients were included in the final analysis, as presented in the CONSORT flow diagram (Figure 1).

Patients received a total volume of 5 mL of anesthetic solution prepared in a single syringe as follows:

I Group (R+D group)—30 patients received 4 mL of local anesthetic 0.5% ropivacaine chloride (ROPIvacaine 5 mg/mL, B|Braun Melsungen AG, 34209 Melsungen, Germany), and 1 mL/4 mg dexamethasone (Dexason^®^, solution for injection, 4 mg/1 mL, GALENIKA a.d., 11080 Belgrade, Serbia);

II Group (R group)—30 patients received 5 mL of local anesthetic 0.5% ropivacaine chloride (ROPIvacaine 5 mg/mL, B|Braun Melsungen AG, 34209 Melsungen, Germany);

III Group (B group)—30 patients received 5 mL of local anesthetic 0.5% bupivacaine (Marcaine^®^, 0.5% Astra Zeneca, Sodertalje, Sweden).

#### 4.1.1. Inclusion and Exclusion Criteria

Participant selection was based on clinical examination, review of medical records, and data obtained through a questionnaire administered to the participants.

The inclusion criteria were as follows: patients scheduled for surgical extraction of impacted mandibular third molar for the first time, diagnosed as horizontal impaction according to Winter’s classification; patients classified as American Society of Anesthesiologists (ASA) physical status I (ASA I) [49]; absence of pain, swelling, or trismus during the two weeks prior to surgery; absence of systemic infection or pericoronitis; no known allergies to local anesthetics, analgesics, or dexamethasone; and no antibiotic therapy within 14 days prior to the surgical removal of the impacted mandibular third molar teeth.

The exclusion criteria were as follows: patients classified as ASA II–VI according to the American Society of Anesthesiologists classification; patients with systemic diseases; individuals who had experienced major stressful life events within the previous six months (e.g., death of a close family member. divorce. or job loss); patients engaged in high-stress occupations and shift work; smokers; patients who had used or were currently using psychoactive medications, sedatives, anxiolytics or antidepressants; corticosteroid therapy, pregnant or breastfeeding women; and women using oral contraceptive pills.

#### 4.1.2. Data Collection

The study was conducted at a single center, the Clinic of Dental Medicine, Department of Oral Surgery, Faculty of Medicine, University of Niš, Niš, Serbia. The data collection process involved obtaining basic information, assessment of the degree of mandibular third molar impaction, evaluation of preoperative stress levels, saliva sampling, and measurement of salivary cortisol levels before and after surgery. The admission and preoperative treatment of all participants started at 8:00 a.m.

#### 4.1.3. Baseline Data Collection

Baseline data collection was performed using a structured questionnaire and included the collection of basic demographic characteristics, general health status, and medication use among the participants.

#### 4.1.4. Assessment of the Degree of Mandibular Third Molar Impaction

The assessment of the degree and position of impaction of the mandibular third molar was performed based on the analysis of panoramic radiographs (orthopantomograms) or CBCT, using Winter’s classification.

#### 4.1.5. Saliva Sampling and Measurement of Salivary Cortisol Levels

The primary outcome measure was perioperative salivary cortisol concentration (ng/mL), assessed as a biomarker of hypothalamic–pituitary–adrenal (HPA) axis activation and perioperative stress response. Saliva samples were collected at three predefined time points: 15 min before surgery, 15 min after surgery, and 24 h postoperatively. Salivary cortisol concentrations were determined using a competitive enzyme-linked immunosorbent assay (ELISA) according to the manufacturer’s instructions under standardized laboratory conditions.

Patients were instructed to avoid strenuous physical activity on the day before the procedure, refrain from any dental interventions, and avoid consumption of foods and beverages high in sugar or caffeine (e.g., sweets, coffee, soft drinks, alcohol) for at least 12 h prior to surgery. On the day of the procedure, participants were required to refrain from brushing their teeth, eating, drinking, using toothpicks, and engaging in physical exertion for at least 60 min before saliva collection. Preparation for the surgical intervention lasted approximately 30 min, during which patients remained in a quiet room. Upon entering the operating room, patients were allowed to rinse their oral cavity with water several times over a period of up to 10 min. Fifteen minutes after the final rinse, a sample of unstimulated mixed saliva was collected from the contralateral, non-operative sublingual region using sterile Pasteur pipettes. Saliva samples were collected between 8:00 and 9:00 a.m. in Eppendorf tubes, temporarily stored at −20 °C for up to 30 min, and then transported to the Scientific Research Center for Biomedicine, Faculty of Medicine, University of Niš, where they were stored at −80 °C until further analysis. Exceptional care was taken to ensure that the saliva was not contaminated with foreign material and blood. The cortisol level in the collected samples was determined via the ELISA method (enzyme-linked immunosorbent assay) using a commercial kit (Cortisol Parameter Assay Kit) following the manufacturer’s instructions (R&D Systems, Biotechnology Company, Minneapolis, MN, USA). To achieve greater sensitivity and specificity of the method, ELISA kits were used, whose analyte detection method is based on the principles of competition. According to this principle, an unknown amount of cortisol in the sample and a known amount of enzyme-conjugated cortisol compete for the same binding sites on antibodies previously adsorbed to the walls of microtiter plate wells (half-microspheres). After the incubation period has ended, the wells (half-microspheres) are washed to stop the competitive reaction, and then the chromogenic substrate is added. The action of the enzyme on the substrate leads to the development of color, whose intensity is determined by the colorimetric method. The intensity of the obtained color is inversely proportional to the concentration of cortisol in the sample, which is determined by reading from the curve constructed based on standards, i.e., solutions of known, increasing concentrations of cortisol.

The measuring range of cortisol concentrations of the kits used ranged from 0.75 μg/mL to 50 μg/mL. The procedure was carried out as follows: After thawing at room temperature, the saliva samples were vortexed and then centrifuged at 3000 rpm for 5 min to precipitate the mucins contained in the saliva. In total, 50 μL of supernatant was collected and diluted at a ratio of 1:5 with the intended diluent (Calibrator Diluent RD5-43). Such prepared samples in a volume of 100 μL were applied to the wells of a microtiter plate whose walls were coated with goat anti-mouse IgG antibodies. Then, 50 μL of horseradish peroxidase-conjugated cortisol (HRT) was added, as well as 50 μL of primary antibody solution (cortisol-specific mouse 111 monoclonal antibody). This was followed by incubation on an orbital mixer (500 rpm) for 2 h at room temperature. During incubation, cortisol from the sample and added cortisol conjugated with HRT compete for the same binding sites on the primary antibody, which in turn binds to antibodies fixed to the walls of the “wells” (half-microspheres) of the microtiter plate.

To remove unbound components, a total of four washings of the plate were carried out on an automatic washer (Biochrom Asys Atlantis Microplate Washer, Biochrom Ltd., Cambridge, UK) with 400 μL of solution per well (Wash Buffer). After washing, 200 μL of substrate (tetramethylbenzidine) was added, followed by incubation for 30 min at room temperature in the dark to allow HRP to react with the chromogenic substrate and enable color development. Color development was stopped by adding 50 μL of 2 N sulfuric acid solution (*Stop Solution*). The optical density of the obtained color was read using the ELISA reader (Multiskan Ascent 354 Microplate Photometer, Thermo Labsystems, Waltham, MA, USA) at a wavelength of 450 nm, with a correction at a wavelength of 540 nm. Based on the optical densities of the applied standards, a standard curve was constructed in a computer program (TableCurve2D, AISN software, USA, ver. 4.0.0.0) in which the cortisol concentrations of the samples were read in ng/mL and then multiplied by 5 due to the initial dilution.

#### 4.1.6. Injection Techniques for Local Anesthetics and Dexamethasone

Mandibular block anesthesia was performed identically in all groups using the Gow-Gates technique [50], targeting the condylar neck, with aspiration prior to injection. Sterile, single-use 5 mL plastic syringes (Nipro syringes, Shanghai International Holding Corp. GmbH/Europe, Eifestrasse 80, 20537 Hamburg, Germany), as carriers for local anesthetics (with verified expiration dates), were used in all groups. A 21 G (0.8 × 40 mm) needle (Nipro needle, Nipro Europe N.V., Weihoek 3H, B-1930 Zaventem, Belgium) was utilized for delivery of the anesthetic to the target site.

For mandibular conduction anesthesia, the following agents were administered: I Group (R+D group)—30 patients received 4 mL of local anesthetic 0.5% ropivacaine chloride (ROPIvacaine 5 mg/mL, B|Braun Melsungen AG, 34209 Melsungen, Germany), and 1 mL/4 mg dexamethasone (Dexason^®^, solution for injection, 4 mg/1 mL, GALENIKA a.d., 11080 Belgrade, Serbia); II Group (R group)—30 patients received 5 mL of local anesthetic 0.5% ropivacaine chloride (ROPIvacaine 5 mg/mL, B|Braun Melsungen AG, 34209 Melsungen, Germany); and III Group (B group)—30 patients received 5 mL of local anesthetic 0.5% bupivacaine (Marcaine^®^, 0.5% Astra Zeneca, Sodertalje, Sweden).

The onset of anesthesia was assessed by the absence of a nociceptive response to gentle probing of the oral and vestibular mucosa of the mandible using blunt instruments (dental surgical forceps and tweezers) and lip numbness on the side of operation.

#### 4.1.7. Surgical Procedure

All patients underwent standardized surgical extraction of impacted mandibular third molars. Extraoral antisepsis was performed using 10% iodine, followed by intraoral rinsing with 0.12% chlorhexidine (20 mL for 3 min). A buccal approach was used with a linear incision (scalpel #15) and a vertical releasing incision, followed by elevation of a mucoperiosteal flap. Bone removal around the crown was performed using a sterile round bur (#167-141, Meisinger HM, Neuss, Germany) under continuous irrigation with cold (8 °C) sterile 0.9% saline. Tooth sectioning and extraction were completed, followed by socket irrigation, flap repositioning, and suturing with 5–0 polyglycolic acid sutures (Marline rapide USP 5/0, Catgut GmbH, Markneukirchen, Germany), which were removed on postoperative day 7. Operative time was recorded from local anesthetic administration to completion of suturing and placement of compressive gauze.

Postoperative treatment included amoxicillin/clavulanic acid (1 g twice daily for 7 days) or clindamycin (600 mg twice daily in penicillin-allergic patients), along with ibuprofen 400 mg twice daily for analgesia.

### 4.2. Secondary Outcome

Secondary outcome measures included preoperative psychological stress, duration of conduction anesthesia, duration of postoperative analgesia, and postoperative analgesic consumption.

#### 4.2.1. Assessment of Preoperative Stress

Preoperative stress subjective assessment, as an important factor influencing patient response to surgical procedures, was evaluated using the Revised Dental Anxiety Scale [51]. Patients completed the questionnaire 30 min before the intervention, while waiting in the waiting area outside the operating room. The Revised Dental Anxiety Scale comprises four items, each scored on a 5-point scale, yielding a total score ranging from 4 to 20. Higher scores indicate greater levels of dental anxiety. Anxiety levels were categorized as follows: 4 = normal reaction, 5–8 = mild anxiety, 9–12 = moderate anxiety, 13–14 = high anxiety, and 15–20 = severe anxiety or phobia. The resulting scores were recorded in the patient research form, providing a measure of preoperative psychological stress for subsequent correlation with physiological markers and perioperative outcomes.

#### 4.2.2. Duration of Conduction Anesthesia

The duration of conduction anesthesia was defined as the interval between the initial onset of the lower lip numbness and its complete resolution (in minutes).

#### 4.2.3. Duration of Analgesia

The duration of analgesia was defined as the time from completion of the surgical procedure to the onset of the first postoperative pain requiring analgesic administration (in minutes).

#### 4.2.4. Postoperative Analgesic Parameters

Postoperative analgesic parameters were evaluated, including the time to first analgesic intake (min) and the total number of analgesics consumed within 24 h following surgery.

### 4.3. Determination of Sample Size

The sample size was calculated based on the primary endpoint, the change in cortisol level between dental extraction procedures derived from the study Gadicherla et al. [24]. Based on these data, a small to medium effect size (f = 0.2) and a type I error (α) of 0.05 for two-way testing of the null hypothesis, study power = 0.8, correlation among repeated measures = 0.5 and a nonsphericity correction ε = 0.75, using rmANOVA design within-between interaction in the program package G power 3.1.9.2, the required sample size was 66 participants (approximately 22 per group). To increase statistical significance, 30 participants were included in each group (total n = 90).

### 4.4. Statistical Analysis

The results regarding salivary cortisol were statistically processed and analyzed using the statistical program SPSS (version 15.0; SPSS, Chicago, IL, USA). Data are presented in tables and graphs with the arithmetic mean and standard deviation, i.e., in the form of absolute and relative numbers. The data distribution was estimated by using the Shapiro–Wilk test.

Statistical analysis was performed using analysis of variance (ANOVA) to assess intergroup differences and repeated measures ANOVA (rmANOVA) to evaluate the effects of group, time, and their interaction on normally distributed data. In rmANOVA sphericity was evaluated using Mauchly’s test. The Greenhouse–Geisser correction was used when the sphericity was violated. When significant differences were detected, appropriate post hoc tests were applied. Comparisons among three or more groups for non-normally distributed or heteroscedastic data were performed using the Kruskal–Wallis (KW) test. The chi-squared test was used for categorical data. The null hypothesis was tested using a significance threshold of *p* < 0.05. No outliers were excluded from the analyses.

## 5. Conclusions

The combination of ropivacaine and dexamethasone significantly reduced perioperative salivary cortisol levels compared to ropivacaine or bupivacaine alone during impacted mandibular third molar surgery. These findings suggest potential attenuation of neuroendocrine response as the results of dexamethasone-induced suppression of hypothalamic–pituitary–adrenal axis activity and improved analgesic effect of this combination. The observed reduction in cortisol levels may contribute to improved physiological stability and postoperative recovery, supporting the potential clinical benefit of this anesthetic approach in oral surgery.

## Figures and Tables

**Table 1:** Baseline demographic and clinical characteristics of study participants by group.

Characteristics	Ropivacaine + Dexamethasone Group (R +D) Mean ± SD	Ropivacaine Group (R) Mean ± SD	Bupivacaine Group (B) Mean ± SD	*p* ^1^
**Age (years) (mean ± SD)**	30.40 ± 6.32	26.27 ± 4.85	29.67 ± 9.68	0.067 ^1^
**Gender**				
Male (n; %)	16; 53.3%	12; 40%	12; 40%	0.488 ^2^
Female (n; %)	14; 46.7%	18; 60%	18; 60%	
Surgery duration (min)	29.82 ± 7.10	33.59 ± 6.32	34.06 ± 4.74	0.098
Revised Norman Corah Dental Anxiety Scale	8.80 ± 1.19	8.83 ± 1.44	8.53 ± 1.70	0.890
Duration of anesthesia (minutes; mean ± SD)	489.13 ± 231.67	273.93 ± 57.97 ^a^	333.13 ± 116.58 ^a,b^	<0.001 ^3^
Number of patients who used analgesics (n; %)	25; 83.3%	30; 100%	29; 96.7%	0.044 ^4^
Duration of analgesia when analgesics were used for the first time (minutes; mean ± SD)	544.21 ± 206.39	284.10 ± 77.98 ^a^	351.21 ± 153.84 ^a^	<0.001 ^3^
Number of analgesics used during 24 h postoperatively (minutes; mean ± SD)	1.96 ± 0.62	3.03 ± 0.67 ^a^	2.29 + 0.98 ^b^	<0.001 ^3^

^1^ ANOVA; ^2^ chi-squared test, ^3^ Kruskal–Wallis’s test, ^4^ Fisher’s test, ^a^ *p* < 0.05 vs. R+D group, ^b^ *p* < 0.05 vs. R group.

**Table 2:** Perioperative salivary cortisol levels in study groups.

Salivary Cortisol Level (ng/mL)	Ropivacaine + Dexamethasone Group (R +D) Mean ± SD	Ropivacaine Group (R) Mean ± SD	Bupivacaine Group (B) Mean ± SD	*p* ^1^
15 min before the surgery	19.45 ± 8.24 ng/mL	18.99 ± 10.19 ng/mL	14.17 ± 9.29 ng/mL	0.984
15 min after the surgery	9.31 ± 7.37 ng/mL	20.56 ± 12.39 ^a^ ng/mL	19.64 ± 12.39 ^a^ ng/mL	0.002
24 h after the surgery	7.13 ± 3.92 ng/mL	15.95 ± 11.80 ^a^ ng/mL	18.1 ± 10.65 ^a^ ng/mL	0.001
***p*** **^2^**	Time effect: 0.004	Interaction: *p* < 0.001	Group effect: 0.005	Partial eta squared 0.200

SD—standard deviation; ^1^ Kruskal–Wallis (KW) test; ^2^ repeated-measures ANOVA; ^a^ *p* < 0.05 vs. R group.

## Data Availability

The original contributions presented in this study are included in the article/Supplementary Materials. Further inquiries can be directed to the corresponding authors.

## Supplementary Materials

The following supporting information can be downloaded at: https://www.mdpi.com/article/10.3390/ph19060930/s1, Supplementary File S1. CONSORT 2010 checklist. Reference [48] is cited in the Supplementary Materials.

## Abbreviations

The following abbreviations are used in this manuscript:

R+D	Ropivacaine + dexamethasone
R	Ropivacaine
B	Bupivacaine
NK	Natural killer
IL-6	Interleukin 6
IL-8	Interleukin 8
IL-1 β	Interleukin 1 β
TNF	Tumor necrosis factor
HPA	Hypothalamic–pituitary–adrenal
CRH	Corticotropin-releasing hormone
ACTH	Adrenocorticotropic hormone
GRES	Glucocorticoid response elements
PGE2	Prostaglandin E2
ASA	American Society of Anesthesiologists
KW	Kruskal–Wallis test
